# Parametric imaging of dynamic long-axial-field-of-view PET scans: Technical challenges, statistical insights and clinical applications^☆^

**DOI:** 10.1016/j.zemedi.2026.01.007

**Published:** 2026-02-01

**Authors:** Fengyun Gu, Clemens Mingels, Robert Seifert, Federico Caobelli, Ali Afshar-Oromieh, Axel Rominger, Finbarr O’Sullivan, Kuangyu Shi

**Affiliations:** a School of Mathematics and Physics, North China Electric Power University, Beijing, China; b Department of Nuclear Medicine, Inselspital, Bern University Hospital, University of Bern, Bern, Switzerland; c School of Mathematical Sciences, University College Cork, Cork, Ireland

**Keywords:** Dynamic LAFOV PET, Parametric imaging, Kinetic analysis, Clinical applications, Open-source software

## Abstract

Dynamic long-axial-field-of-view (LAFOV) PET imaging offers unprecedented opportunities for quantitative assessment of tracer kinetics across the entire body. This review discusses the core technical challenges posed by LAFOV datasets in parametric image generation and introduces methodological developments from a statistical perspective, including arterial input function strategies, classical and flexible kinetic models (compartment models, spectral analysis, adiabatic approximation to the tissue homogeneity, and non-parametric models), graphical techniques (Patlak, Logan, and their variants), and emerging directions such as direct parametric reconstruction, dimension reduction, and deep learning. These methodologies are further linked to dynamic clinical protocols designed to shorten scanning times, enable multi-tracer injections, support low-dose imaging, and open avenues for novel tracer and drug development. Finally, we summarize the most recent software packages, particularly those tailored for LAFOV PET parametric imaging. These advances indicate that reliable parametric imaging holds promise for broader clinical adoption, grounded in robust modeling, multi-center validation, and ongoing software advancements.

## 1 Introduction

Positron emission tomography (PET) is a powerful molecular imaging modality that enables the visualization and quantification of physiological and pathological processes in vivo, widely used in oncology, neurology, and cardiology. Recently developed long-axial-field-of-view (LAFOV) PET/CT scanners, exemplified by the uEXPLORER (United Imaging Healthcare) [1], PennPET Explorer (University of Pennsylvania) [2], Biograph Vision Quadra (Siemens Healthineers) [3], uMI Panorama GS [4] and Omni (GE HealthCare) are increasingly being installed at medical centers worldwide. Such innovations, characterized by ultra-high sensitivity (10∼40 times), extended field of view (1∼2 m), and enhanced temporal resolution (0.1 s), facilitate simultaneous imaging of the entire human body or major organ systems in a single bed position. Over the past several years, nearly 400 publications have explored or discussed diverse clinical applications of LAFOV PET, with a particular emphasis on static imaging protocols, such as rapid scanning, low-dose radiotracer administration, and delayed imaging [5], [6], [7], [8].

Compared with a single static snapshot, dynamic PET captures the full time course of radiotracer uptake and distribution, providing spatiotemporal four-dimensional (4D) datasets. Dynamic LAFOV studies have been reported using a wide range of radiotracers, spanning multiple clinical focus areas. For example, ^18^F-FDG is commonly used for oncologic applications, including tumor detection and characterization [9], [10]. Tracers such as ^15^O-H${}_{2}$O, ^82^Rb and ^11^C-Butanol have been employed for quantitative myocardial blood flow assessment in cardiovascular diseases [11], [12], [13]. Neurological applications have also been explored, with ^11^C-CFT used to investigate Parkinson’s disease [14]. Representative clinical applications of dynamic LAFOV PET across these focus areas are summarized in Table 1, which provides an overview of currently available LAFOV PET scanners, vendors, axial-field-of-view (AFOV), participating institutions and locations, as well as reported dynamic datasets acquired on these systems. As ^18^F-FDG is routinely used in nearly all clinical PET installations, the table only focuses on non-FDG tracers. For specific acquisition protocols and details, we refer readers to our previous review [15].

Building on the availability of diverse dynamic datasets acquired on LAFOV PET systems, quantitative analysis of dynamic PET data plays a central role in extracting physiologically meaningful information. By applying appropriate methods at the voxel level, physiological parameters like blood flow, metabolism, and receptor-binding can be estimated to yield parametric images that map these kinetics across the entire field of view [16]. Relative to conventional standardized uptake value (SUV) measurements, parametric imaging offers great potential to improve diagnostic accuracy, support personalized treatment planning, and monitor therapeutic responses, particularly for complex diseases [17].

Despite growing enthusiasm for LAFOV parametric imaging, the literature remains limited [49]. A PubMed search excluding review papers and commentaries identified only over 40 relevant papers. Most studies still rely on standard two tissue compartmental modeling (2C) or Patlak analysis, with preliminary efforts to refine these approaches [50]. However, dynamic LAFOV datasets present significant challenges, including substantial variability in tracer kinetics across tissues and the computational demands of processing large voxel volumes. Addressing these issues requires advanced quantitative techniques and dedicated software tools to fully release the potential of LAFOV parametric imaging [51]. Another key obstacle to clinical adoption of parametric imaging is the long acquisition time of dynamic scans, a challenge that is even more pronounced in multi-tracer protocols.

**Table 1:** Overview of LAFOV PET scanners, vendors, axial-field-of-view (AFOV), institutions, and representative non-FDG tracers used in dynamic imaging across major clinical focus areas.

PET Scanner	Vendor	AFOV (cm)	Institution/Medical center	Tracer	Focus area
uEXPLORER	United Imaging Healthcare	194	Zhongshan Hospital, Shanghai, China	^68^Ga-DOTATATE [18], [19], [20]	Oncology
			Renji Hospital, Shanghai, China	^68^Ga-FAPI-04 [21], [22], [23]	Oncology
				^68^Ga-PSMA-11 [24], [25]	Oncology
				^11^Cmethionine [26]	Oncology
				^11^C-CFT [14]	Neurology
			Peking University Cancer Hospital, Beijing, China	^18^F-AIF-NOTA-HER2-BCH &	Oncology
				^18^F-AIF-RESCA-HER2-BCH [27]	
				^68^Ga-N188 [28]	Oncology
				^68^Ga-Ga-HNI01 [29]	Oncology
				^68^Ga-NC-BCH [30]	Oncology
				^68^Ga-Ga-NOTA-SGC8 [31]	Aptamer
				^68^Ga-Ga-B7H3-BCH [32]	Oncology
			Nanfang Hospital, Guangzhou, China	^18^F-FAPI-42 [33]	Oncology
				^18^F-FAPT & ^18^F-FAPI-42 [34]	Oncology
				^18^F-FAPTG [35]	Oncology
			Guangdong Provincial People’s Hospital, Guangzhou, China	^18^F-PSMA-11 [36]	Oncology
			University of California Davis, California, USA	^18^F-Fluciclovine [37]	Oncology
				^11^C-Butanol [13]	Cardiology
				^89^Zr-Df-Crefmirlimab [38]	Immunology
				^18^F-NaF [39]	Hematology
				^18^F-PI-2620 [40]	Neurology
				^18^F-AraG [41]	Oncology
				^18^F-florbetaben [42]	Neurology

Quadra	Siemens Healthineers	106	Bern University Hospital, Bern, Switzerland	^82^Rb [12]	Cardiology
				^18^F-FET [43]	Neurology
			Turku PET Center, Turku, Finland	^15^O-H${}_{2}$O [11]	Cardiology
			Aarhus University, Aarhus, Denmark	^15^O-H${}_{2}$O [44]	Oncology
			Amsterdam UMC, Amsterdam, Netherlands	^68^Ga-FAPI-46 [45]	Oncology
				^18^F-DPA-714 [46]	Inflammation
			German Cancer Research Center, Heidelberg, Germany	^18^F-PSMA-1007 [47]	Oncology

PennPET Explorer	University of Pennsylvania	140	University of Pennsylvania, Philadelphia, USA	^18^F-FGln [48]	Oncology

uMI Panorama GS	United Imaging Healthcare	148			

Omni	GE HealthCare	128			

In this review, we focus on the generation of parametric images with the state-of-the-art LAFOV PET scanners. We first introduce the technical challenges inherent to 4D LAFOV data processing, then review current strategies for quantitative analysis, covering: (i) extraction of arterial input function; (ii) dimension reduction and acceleration algorithms; (iii) kinetic models for accurate parameter estimation; (iv) deep learning based methods; and (v) other techniques associated with parametric imaging. Finally, we discuss emerging methods to overcome the prolonged acquisition times of dynamic scanning, including both single- and multi-tracer protocols, and highlight new clinical opportunities brought by LAFOV PET, including ultra-low-dose dynamic imaging and the development of novel tracers and drugs. Available software tools for LAFOV parametric imaging are also summarized.

## 2 Technical challenges

**Fig. 1:**
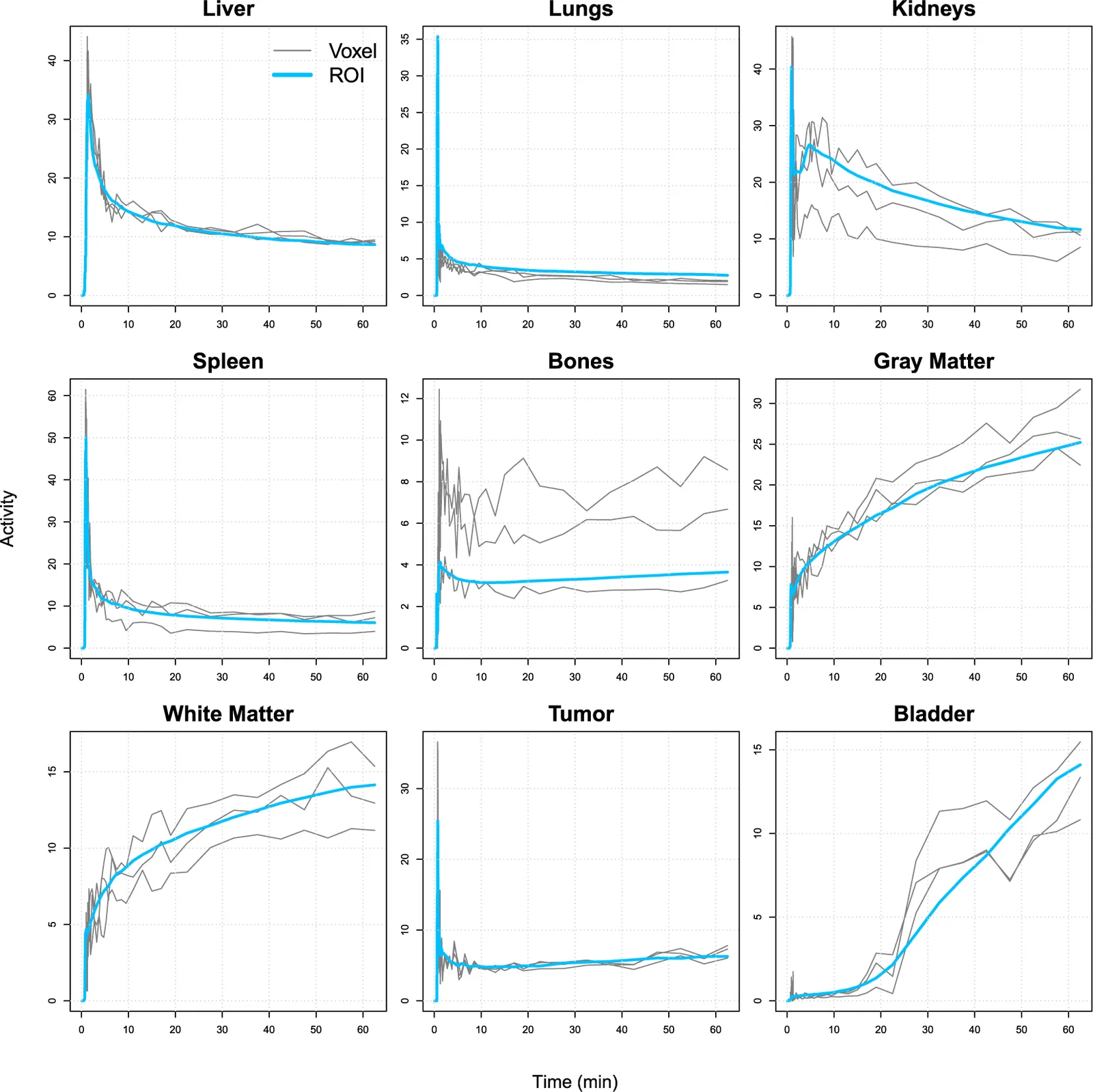
Time activity curves at ROI and voxel levels from a patient injected with ^18^F-FDG and scanned on the Siemens Biograph Vision Quadra PET/CT.

While region of interest (ROI) analysis can only yield physiological parameters for specific tissues, parametric imaging requires quantitative analysis at every voxel level to generate comprehensive maps of kinetic parameters across the entire field of view. Dynamic PET in the LAFOV era, particularly for generating reliable whole-body parametric images, encounters unique obstacles:

(1) Computational bottlenecks: the voxel-by-voxel approach involves processing enormous datasets, with tens of millions of voxels each associated with time activity curves (TACs). This makes voxel-level kinetic modeling computationally demanding, necessitating graphics processing unit (GPU) acceleration and advanced algorithms to handle ultra-large data efficiently [15].
(2) Spatial–temporal correlations: high sensitivity enhances temporal resolution but amplifies correlations across space and time in these high-dimensional (4D) datasets. In conventional parametric imaging workflows, kinetic modeling is typically performed in a voxel-wise manner, treating voxels independently and thus not explicitly accounting for spatial–temporal dependencies between neighboring voxels. When these amplified correlations are ignored, parameter estimation may become unstable or biased, resulting in increased variance, spatially structured artifacts, and a loss of quantitative accuracy in parametric images [52], [53], [54], [55].
(3) Noise management: despite the high sensitivity of LAFOV PET scanners, which reduces overall noise compared to conventional systems, voxel-level TACs still exhibit substantial noise, posing a persistent challenge, particularly in low-count regions or during ultra-short frames, as illustrated in Fig. 1. Thus more precise kinetic models are essential to preserve accuracy without sacrificing detail [56].
(4) Tracer kinetics diversity: TAC shapes vary widely across tissues (Fig. 1), particularly in complex organs like the bladder, liver, and kidneys, where heterogeneous kinetics (e.g., reversible vs. irreversible uptake) prevail. For novel tracers, where underlying physiological mechanisms are often unclear or unknown, standard modeling assumptions may fail, exacerbating challenges in multi-tracer and multi-tissue scenarios [10], [57].
(5) Standardization and data availability: The absence of open-source dynamic LAFOV datasets hinders multi-center validation, benchmarking, and standardization efforts. Differences in acquisition protocols, correction methods, and reconstruction algorithms across centers further hamper reproducibility and cross-study comparisons, limiting the broader adoption of parametric imaging [16].

## 3 Methodologies

**Fig. 2:**
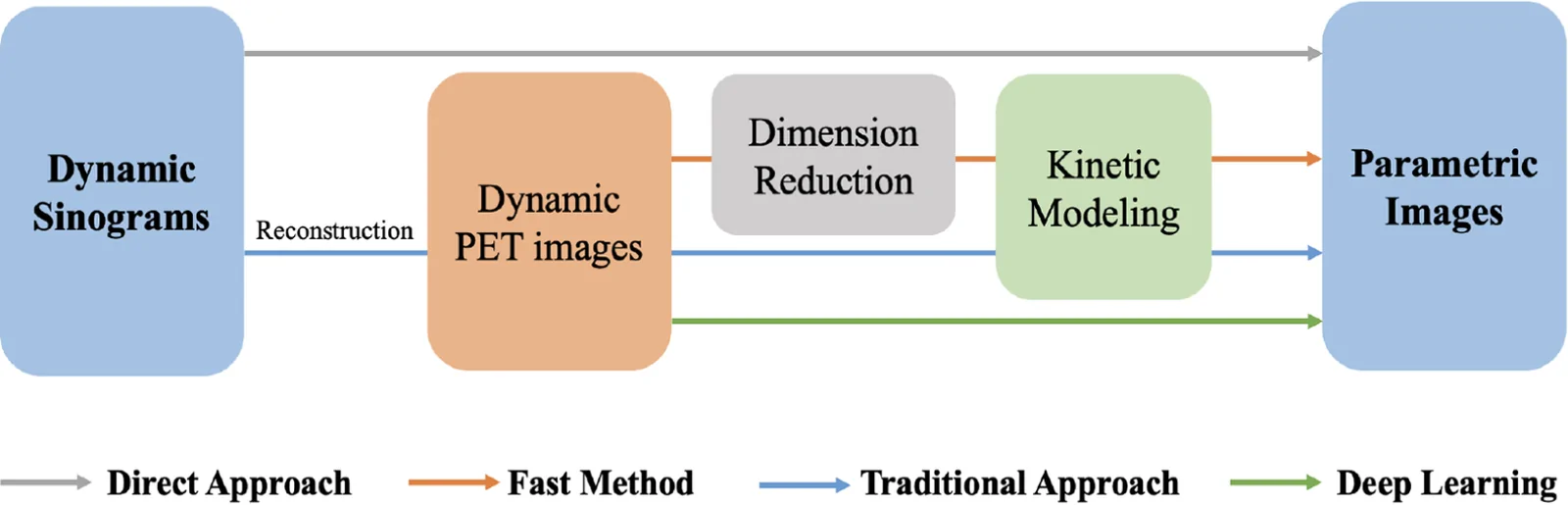
Workflow for the generation of parametric images.

The generation of parametric images in dynamic PET traditionally relies on two primary steps: accurately determining the arterial input function (AIF) and applying suitable kinetic modeling to voxel-level dynamic PET data. This classical framework has provided a solid foundation for tracer kinetic analysis. However, the advent of LAFOV PET scanners, together with advances in computing and algorithmic development, is reshaping this paradigm (Fig. 2). Emerging strategies include:(i) direct parametric reconstruction from list-mode data, which integrates kinetic modeling into the reconstruction process; (ii) dimension-reduction methods to efficiently handle the massive voxel data; and (iii) deep learning approaches capable of generating parametric imaging without the explicit need for AIF or kinetic modeling. These innovations open the way for faster, more robust, and potentially more accurate parametric imaging. We will describe these techniques separately in the following sections, emphasizing their strengths and limitations in the context of LAFOV parametric imaging. For rigorous mathematical derivations, readers are referred to our previous work [15], [51].

### 3.1 Arterial input function

To generate parametric images, the knowledge of the tracer’s arterial plasma concentration is essential, serving as the arterial input function (AIF). Arterial blood sampling has traditionally been regarded as the gold standard for obtaining this input function. However, this method is invasive, causing patient discomfort (e.g., through arterial line insertion and increased radiation exposure) and incurring additional costs for analyzing blood samples. In practice, acquiring arterial blood samples is even more challenging in LAFOV PET studies, as access to the radial artery and antecubital vein is hindered by the extended scanner coverage. Currently, only a very limited number of institutions perform arterial blood sampling. The first attempt was reported by a Danish group, who successfully obtained arterial blood samples for ^15^O-H${}_{2}$O scanning with Quadra [58]. More recent studies have involved ^18^F-FDG [59] and ^68^Ga-FAPI-46 tracers [60] on the Quadra at Amsterdam UMC.

The mainstream non-invasive approaches for estimating AIF include: (i) population-based input function (PBIF) and (ii) image-derived input function (IDIF). PBIF is more commonly used in short-scan LAFOV protocols, for more details, see Section 4.1. IDIF is obtained from a ROI delineated over the blood pool directly on PET images. The high spatial and temporal resolutions of LAFOV PET scanners result in more accurate and less noisy IDIFs, offering multiple options such as the left ventricle, aorta, and other large blood vessels. Comparisons of candidate ROIs have been conducted separately on individual LAFOV platforms, including uEXPLORER [61], [62], Quadra [10], [63], and PennPET Explorer [64]. These scanner-specific evaluations have led to the ROI over descending aorta (DA) becoming the most commonly adopted one for IDIF extraction. For a comprehensive review, we refer readers to [65]. Here we focus solely on discussing several key issues associated with IDIF in LAFOV studies and highlight the recent technical solutions that have been developed:

(1) Delay: In routine PET imaging, the IDIF is typically extracted from an arterial blood pool located close to the target tissue, so the time delay is very short and often negligible. In LAFOV studies, however, the IDIF may be derived from vascular regions located far from some tissues. The resulting delay can reach up to 50 s and may vary considerably across different tissues, making it a critical factor influencing kinetic quantification. To correct for this effect, the delay term is commonly estimated jointly with other parameters of interest using a grid search scheme, which substantially increases the computational burden of the estimation procedure [50], [57]. Two main strategies have been proposed to improve the efficiency of delay estimation: (i) determining the delay within a simplified kinetic model [66], [67], [68] or (ii) applying pulse timing based methods [69].
(2) Dispersion: As another factor that may influence kinetic accuracy, dispersion has received particular attention in tracers with very fast kinetics, especially those focused on the accurate quantification of cerebral blood flow [70], [71]. Dispersion may also occur when the tracer travels from the vascular region used to derive the IDIF to downstream capillary beds, such as those in the lungs. However, corrections for dispersion have rarely been investigated in LAFOV PET studies. To date, only Wang et al. have reported a study demonstrating that modeling both delay and dispersion improves model fitting and has a significant impact on quantitative results in the lungs of healthy subjects [72].
(3) Dual-blood supply: certain organs, e.g., liver, receive dual blood supplies from the hepatic artery and portal vein. Rainio et al. proposed a novel compartment model that accounts for the influence of dual blood supplies, enabling hepatic blood flow quantification in humans from ^15^O-H${}_{2}$O PET images [73]. Wang et al. also demonstrated the effects of dual blood supply in the lungs using their proposed dual-blood input function (DBIF) model [74].
(4) Partial volume effect: despite the improved spatial resolution of LAFOV PET scanners, partial volume effects in IDIFs cannot be completely avoided and still may lead to biased estimation of tracer input functions and subsequent kinetic parameters. While partial volume correction (PVC) methods have been extensively investigated in conventional PET systems [75], to the best of our knowledge, dedicated and systematic studies focusing on PVC for IDIF extraction in LAFOV PET have not yet been reported.
(5) Metabolites: reliable quantitative results can be obtained with radiotracers that do not produce metabolites, such as ^18^F-FDG, and are also achievable for tracers that form metabolites, provided that the input function is properly corrected for metabolite contributions. As shown in Table 1, many non-FDG tracers have been investigated on LAFOV PET scanners, and some of them are metabolized, generating circulating metabolites [76]. To account for these metabolites, in addition to conventional chemical approaches, there is a need to develop data-driven methods for estimating the metabolite fraction [77], [78]. However, such data-driven metabolite correction methods have not yet been reported or systematically evaluated in the context of LAFOV PET imaging.

### 3.2 Dimension reduction algorithms

Dynamic LAFOV PET datasets are enormous, typically on the order of tens of gigabytes, with the number of voxels exceeding hundreds of millions. For relatively simple and noise-robust models (e.g., Patlak analysis), parametric imaging can still be generated by independently fitting voxel TACs. However, for more complex models (e.g., 2C model), such a fitting strategy substantially increases the computational burden. Current approaches therefore primarily rely on GPU acceleration to speed up the estimation process [67], [79], [80],while lacking algorithmic innovations for effectively handling the scale and dimensionality of LAFOV PET data.

From a methodological perspective, the analysis of dynamic LAFOV PET data is inherently a multivariate statistical problem. A variety of statistical methods, including factor analysis (FA) [81], singular value decomposition (SVD) [82], and principal component analysis (PCA) [83], [84], have been applied to perform spatio-temporal analysis of conventional PET data. These approaches enable effective data dimension reduction, improving computational efficiency, while simultaneously suppressing spatially structured artifacts, and preserving quantitative accuracy in parametric images [52], [85], [86], [87].

Building on the mixture model framework originally proposed by O’Sullivan in 1993 [88], our recently developed non-parametric residue mapping (NPRM) algorithm [89], [90] has been successfully applied to the dynamic LAFOV PET, enabling faster and more accurate parametric image generation [57]. In the mixture model, the voxel-level TAC, $z_{i}\left(t\right)$, is expressed as a non-negative linear combination of sub-TACs ($u_{k}$):

(1) $$z_{i}\left(t\right)=\alpha_{1}\mu_{1}\left(t\right)+\cdots \alpha_{K}\mu_{K}\left(t\right),i=1....N,t=1...T,$$

where α denotes the spatial coefficient, N and T are the number of voxels and time frames, respectively. $\left\{\mu_{1},\ldots ,\mu_{K}\right\}$ represent distinct kinetic patterns obtained by segmenting the dynamic PET data. Unlike segmentation of static PET images, this approach defines segments based on voxel-level TAC characteristics, not anatomical structures.

Recently Turku PET Centre has reported relevant work on evaluation of several commonly used clustering methods in PET images. Their findings indicate that independent component analysis (ICA), Gaussian mixture model (GMM), and fuzzy c-means (FCM) combined with mini-batch K-means hold promise for dynamic LAFOV PET segmentation [91], [92]. Through this process, kinetic analysis is subsequently performed only on the TAC of each segment, rather than voxel by voxel. The parameters of interest are then mapped back to the image domain using the spatial coefficients. This substantially accelerates the generation of parametric images. However, this area of research remains relatively less studied, and further methodological developments are highly desirable in the future.

### 3.3 Kinetic models

Assuming the radiotracer’s interaction with tissue is linear and time-invariant, the measured TAC can be expressed as the convolution of the arterial input function ($C_{p}$) and the tissue impulse response function (R):

(2) $$z\left(t\right)=R\left(t\right)⊗C_{p}\left(t\right),$$

where z(t) represents the TAC of a specific voxel, segment or ROI. Given a known input function, the core task of kinetic modeling lies in estimating the response function and deriving associated kinetic parameters, including blood volume ($V_{B}$), flow (K), influx ($K_{i}$) and volume of distribution ($V_{D}$) [16], [93].

**Fig. 3:**
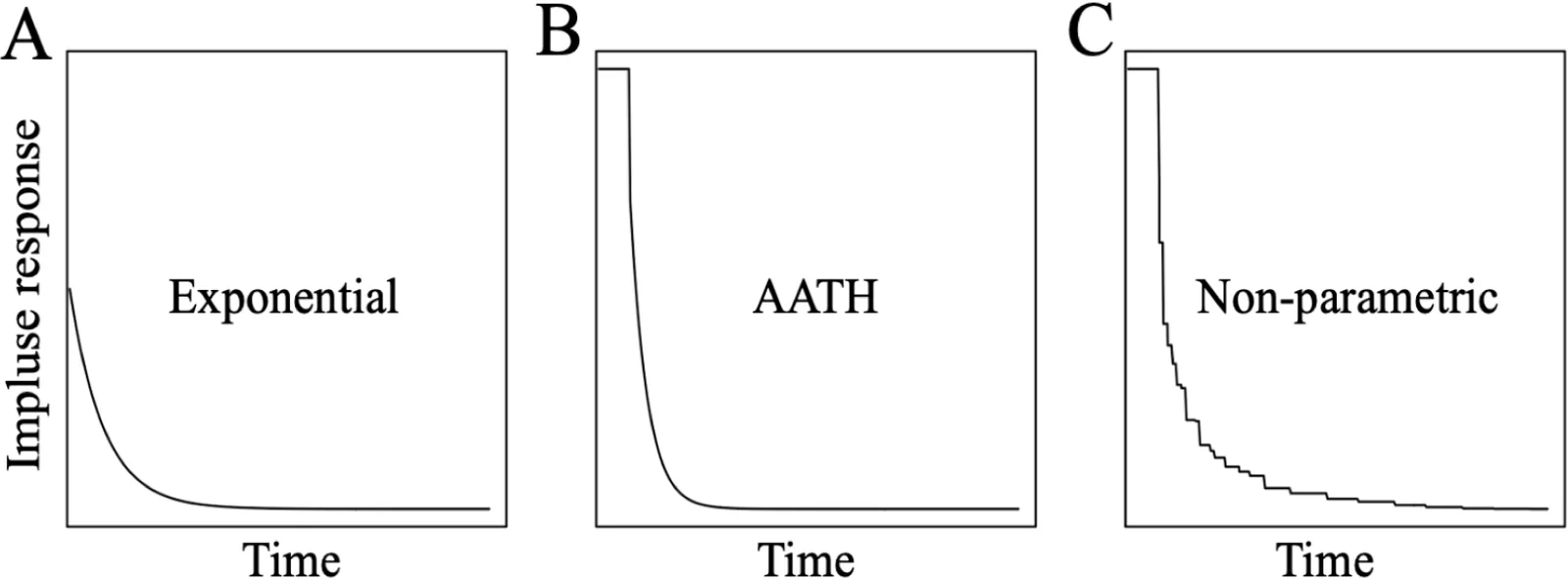
The representative response functions R(t) in compartmental (exponential from), AATH (adapted exponential form) and non-parametric models (piecewise-linear form).

The essential differences among kinetic models stem from assumptions about the response function, particularly its shape (as illustrated in Fig. 3). Traditional kinetic models typically assume an exponential form, but this may not always hold in the complex and heterogeneous physiological environment of LAFOV PET studies [15]. Recent research has therefore relaxed this constraint [57], [94], adopting adapted exponential forms or even completely non-parametric approaches that do not impose an exponential shape (see the summary in Table 2). In the following sections, we will discuss these traditional and emerging models in detail.

**Table 2:** Summary of kinetic models and their assumed response function.

Model	Assumed shape	Number
One-tissue compartmental model	Exponential	One
Two-tissue compartmental model	Exponential	Two
Spectral analysis	Exponential	Adaptive
AATH model	Adapted exponential	One
Non-parametric model	Piecewise-linear/B-Spline	Adaptive

#### 3.3.1 Models based on exponential form assumptions

Classical compartmental models, such as the one-tissue and two-tissue compartmental models (1C and 2C), serve as the foundation for tracer kinetics in dynamic PET data analysis. These models are described by linear, first-order, time-invariant differential equations characterized by rate constants ($K_{1}$ in ml/min/cm${}^{3}$, $k_{2}$, $k_{3}$ and $k_{4}$ in min^−1^). These equations are commonly solved using numerical optimization approaches, most notably nonlinear least squares (NLS), to estimate the impulse response function and the associated kinetic parameters [95], [96], [97], [98], [99]. Several accelerated solution strategies have also been developed to improve computational efficiency, such as basis function methods [44], [100], [101], [102], [103]. Importantly, regardless of the specific solution strategy employed, compartmental models fundamentally assume that the impulse response function follows an exponential form. This modeling assumption represents a key conceptual distinction from the non-parametric approaches introduced in the following sections, which do not impose explicit parametric constraints on the response function.

(1) 1C model: under the exponential form assumption, the tissue response is a single exponential,
  (3) $$R\left(t\right)=K_{1}e^{-k_{2}t}.$$
  It is widely used for quantifying tissue perfusion. The Turku PET centre pioneered dynamic LAFOV PET scanning with ^15^O-H${}_{2}$O, as detailed in [104] and proposed refinements of 1C model that account for vascular delay and organ-specific model selection [102], [103]. Additionally, six variations of 1C have been systematically evaluated for total-body perfusion imaging [105]. For a comprehensive overview of one compartmental modeling approaches, we recommend the systematic review by Rainio et al. [106]. These studies underscore the importance of carefully assessing one compartmental models to determine the most suitable options for total-body perfusion imaging.
(2) 2C model: the tissue impulse response function for 2C model is expressed as a sum of two exponential terms:
  (4) $$R\left(t\right)=K_{1}\left(\pi_{1}e^{-\theta_{1}t}+\pi_{2}e^{-\theta_{2}t}\right),$$
  where $\pi_{1},\pi_{2},\theta_{1},\theta_{2}$ are algebraic functions of $k_{2},k_{3},k_{4}$. The majority of LAFOV dynamic PET scans utilize ^18^F-FDG, with kinetic analyses mainly based on the standard 2C model. This model effectively captures the reversible uptake and phosphorylation of FDG in tissues, enabling detailed quantification of metabolic processes. The impacts of tracer delay, dispersion, motion and optimal model selection have been investigated in recent LAFOV studies [50], [67], [69], [72], [107]. These results emphasize that careful handling of these effects are essential to enhance parametric imaging accuracy for LAFOV ^18^F-FDG studies.
(3) Spectral analysis (SA): proposed by Cunningham and Jones in 1993 [108], SA assumes the response function to be a weighted sum of exponential terms:
  (5) $$R\left(t\right)=\overset{J}{\underset{j=0}{\sum }}a_{j}e^{-\beta_{j}t},a_{j}\geq 0,\beta_{j}\geq 0,\beta_{0}=0.$$
  Here, the eigenvalues $\beta_{j}$ are predefined, while the amplitudes $a_{j}$ are estimated using the NLS algorithm. The model structure, such as reversibility or irreversibility, and the effective number of compartments is inferred from the $a_{j}$ values, known as the spectrum. Although still grounded in exponential forms, SA does not require predefined compartment numbers, enabling the capture of heterogeneous and unknown kinetics. This makes it particularly suitable for LAFOV PET studies and novel tracers. Initial demonstration of SA derived parametric images on LAFOV PET was reported by Boellaard et al. [109], but systematic investigations and adaptations for dynamic LAFOV imaging are still needed.

#### 3.3.2 Models without enforced exponential assumptions

Besides improving traditional models, emerging models have been developed to address the limitations of exponential assumptions in LAFOV PET studies, including the adiabatic approximation to tissue homogeneity (AATH) model and non-parametric approaches. These methods accommodate non-exponential forms, providing greater flexibility for modeling complex tracer kinetics.

**Fig. 4:**
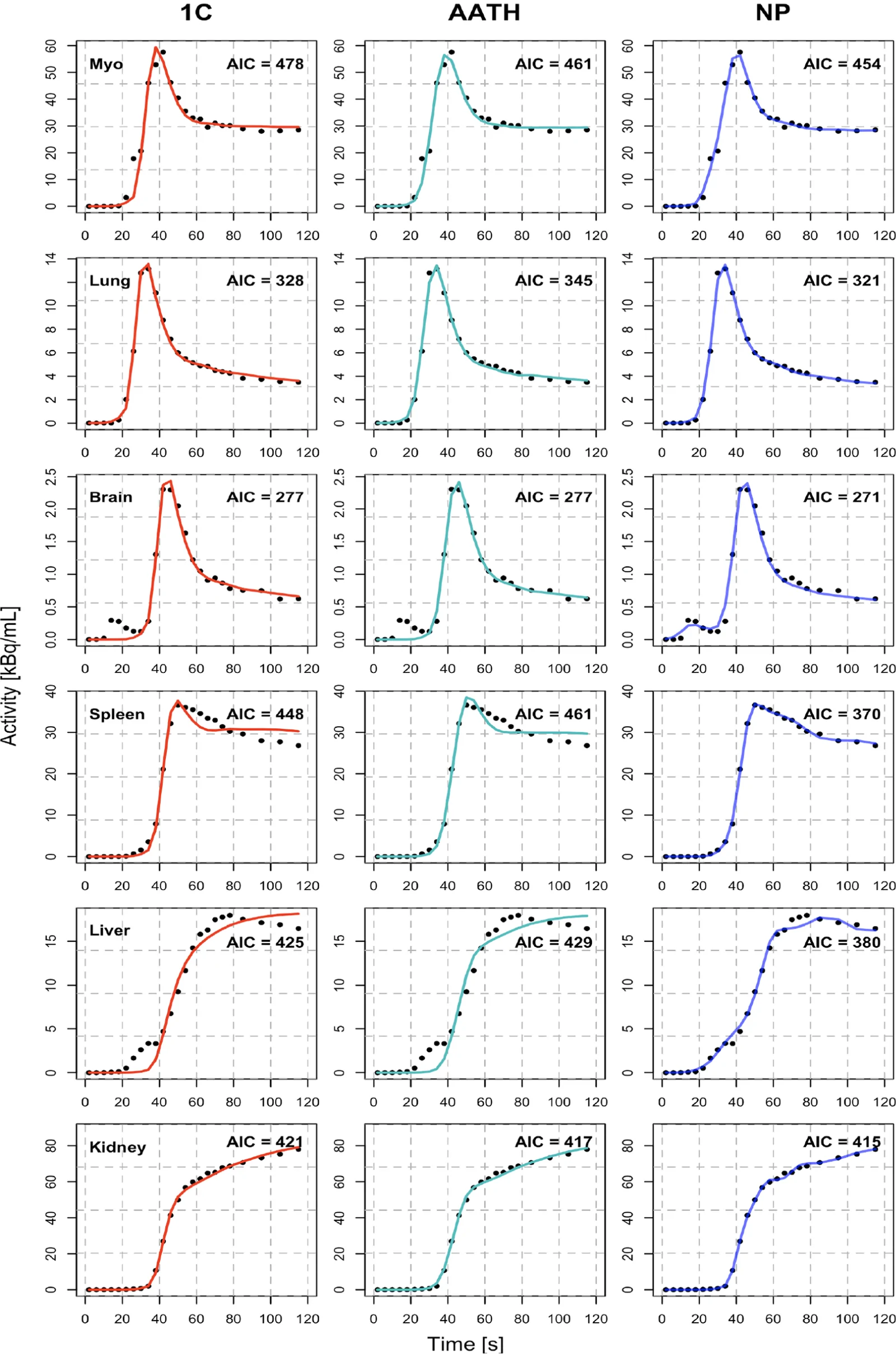
Representative time activity curves from dynamic ^82^Rb-PET imaging acquired on a Siemens Biograph Vision Quadra scanner in six regions: myocardium (myo), lung, brain, spleen, liver, and kidney. Fits are shown from three kinetic models: one-compartment (1C), adiabatic approximation to tissue homogeneity (AATH), and non-parametric (NP) models. Akaike information criterion (AIC) values are provided for each model to evaluate the model performance.

(1) AATH model: in physiological reality, radiotracers require a nonzero transit time to traverse the length of blood vessels at a rate determined by blood flow. This phenomenon can be explicitly accounted for using AATH model, a distributed kinetic model that offers a closed-form time-domain solution [110]. The impulse response function for the AATH model is given by:
  (6) $$R\left(t\right)=\left\{\begin{array}{cc} F,\; & 0\leq t<T_{c},\\ K_{1}e^{-k_{2}\left(t-T_{c}\right)},\; & t\geq T_{c}\;, \end{array}\right)$$
  where F represents the intravascular blood flow and $T_{c}$ is the mean vascular transit time for the radiotracer to pass through the total vascular blood volume. Chung et al. evaluated the performance of the AATH model in quantitative total-body imaging of blood flow using high-temporal-resolution (HTR) early dynamic ^18^F-FDG PET on the uEXPLORER scanner. Their findings demonstrated that the AATH model outperforms the standard 1C model, as assessed by the Akaike information criterion (AIC) [94].
(2) Non-parametric model: in classical exponential-form models, the impulse response is constrained to be monotone decreasing, but the strict monotonicity (ΔR(t)<0) is not always realistic [111]. A priori exponential shape may also be inadequate when in-vivo biochemistry is uncertain [112], [113], [114], [115], especially in emerging LAFOV PET applications. Unlike the parametric approaches above, the residue can be estimated non-parametrically as a nonnegative linear combination of flexible basis functions:
  (7) $$R\left(t\right)=\overset{J}{\underset{j=1}{\sum }}a_{j}I_{j}\left(t\right),\;a_{j}\geq 0,$$
  where I denote generic basis elements, such as B-splines [116], [117] or piecewise functions [89], [118]. Coefficients $a_{j}$ are typically obtained by quadratic programming (QP). This data adaptive formulation can represent both monotone (including exponential) and non-monotone responses without imposing unrealistic parametric restrictions [90]. Our recent work has fully demonstrated that non-parametric (NP) approach can provide more accurate kinetic quantification across multiple tissues compared to 2C model, particularly in metabolically complex organs such as the liver, kidney, and bladder, as shown in ^18^F-FDG PET studies [57]. In addition, our initial findings in whole-body perfusion imaging using ^82^Rb-PET indicate that the NP model consistently outperforms both the AATH and 1C models across multiple organs, as illustrated in Fig. 4.

#### 3.3.3 Graphical analysis

Graphical analysis offers some simple and robust approaches to generate parametric images. It relies on data from a few late time frames of dynamic scanning and employs a non-iterative strategy, ordinary least squares (OLS), to compute the terminal linear slope of transformed TAC.

These approaches are much faster and less sensitive to noise compared to NLS, making it well suited for voxel-level applications, especially in LAFOV PET. However, it often requires specific model assumptions, such as linear relationships in transformed data, and typically yields only a limited set of parametric images, restricting its utility for comprehensive kinetic quantification [119].

As the most widely used graphical approach, the Patlak model estimates $K_{i}$ as the slope of the linear portion of the Patlak plot after an equilibration time ($t^{*}$), and is appropriate for tracers with effectively irreversible trapping (e.g., ^18^F-FDG within a suitable time window) [120]. It has found extensive applications in LAFOV PET parametric imaging. Some adaptations, including generalized Patlak [121], direct Patlak [10], [122], [123], [124], direct generalized Patlak [125], relative Patlak [126], further extend the robustness of Patlak-derived parametric imaging.

For reversible radioligand tracers, Logan plot [127] and relative-equilibrium (RE) plot [128], are routinely used to estimate the total distribution volume ($V_{D}$). These non-iterative regressions can be applied at voxel-level once the required equilibrium conditions are satisfied. For LAFOV PET, preliminary applications have been reported [22], [60], [129], [130], [131], but the literature remains limited, primarily due to the scarcity of dynamic scanning data for such tracers, as detailed in Table 1.

### 3.4 Deep learning

As one of the most active areas of research, deep learning has shown great promise in LAFOV PET studies. Wang et al. provided a systematic review of the potential opportunities for employing deep learning to total-body PET quantification [61]. A few initial efforts have been undertaken to generate parametric images directly, bypassing the need for AIF extraction and the application of kinetic models. Huang et al. explored the potential of direct parametric image generation with the uEXPLORER by using a 3D U-Net convolutional neural network (CNN) framework to train $K_{i}$ images from static SUV data [132]. Wang et al. introduced an improved cycle generative adversarial network (GAN) to learn the mapping relationship between static PET and $K_{i}$ parametric images [133]. Li et al. reconstructed high quality direct Patlak $K_{i}$ images from five-frame sinograms without an input function, using a DeepPET-based framework implemented on Quadra [123].

Deep learning approaches for parametric imaging still face several limitations: (i) the methods employed to date are predominantly supervised learning, which requires establishing ground truth. As discussed above, parametric images generated by either compartmental modeling or the Patlak model are not entirely accurate. Consequently, if the ground truth is flawed, the resulting outputs will undoubtedly be erroneous. (ii) given that LAFOV PET is an emerging technology, the number of available dynamic PET studies for training datasets remains limited, which hampers model generalization and robustness. Moreover, there are currently no open-source dynamic datasets available to facilitate broader research and development. (iii) the data volume for a single patient is substantial, imposing a heavy computational burden and this is particularly exacerbated for methods that aim to train parametric imaging directly from sinograms.

To address some of these challenges, Tehlan et al. proposed a physiological neural representation based on implicit neural representations (INRs) to estimate personalized kinetic parameters. This approach requires minimal training data and alleviates the computational burden [134]. While promising, this direction has not yet been extensively investigated and further work is needed to overcome the above limitations.

### 3.5 Other techniques associated with parametric imaging

#### 3.5.1 Motion correction

Dynamic PET imaging is inherently affected by various sources of motion, and LAFOV PET is no exception. Patient motion, respiratory and cardiac motion, as well as gastrointestinal movement, can all compromise the accuracy of derived parametric images. These challenges underscore the need for sophisticated motion-correction algorithms. Several vendor-provided reconstruction frameworks, such as the United Imaging Reconstruction Toolbox (URT), incorporate motion-correction strategies directly during image reconstruction. A limited number of studies have also investigated motion correction in dynamic whole-body PET imaging [107], [135], [136], [137], but further methodological development and validation remain necessary.

#### 3.5.2 Noise reduction

Noise reduction is a long-standing and essential topic in PET imaging, and it is particularly critical for dynamic PET due to the low count statistics of individual frames. Denoising can be applied either to dynamic images prior to quantitative analysis via post-reconstruction smoothing, or to parametric images directly. Commonly used approaches include classical filters such as Gaussian and non-local-mean (NLM) filters [138], [139], [140], where the trade-off between noise reduction and spatial blurring requires careful statistical consideration. More recently, deep learning–based denoising methods have been introduced for dynamic PET and have shown promising performance [141], [142], [143]. These techniques are expected to be increasingly important for advanced clinical scenarios, including ultra-short dynamic scans (Section 4.1) and ultra-low dose studies (Section 4.3).

#### 3.5.3 Uncertainty quantification

Uncertainty quantification in parametric imaging is essential for assessing the reliability of estimated kinetic parameters and has the potential to further support clinical decision-making. Traditional approaches include methods based on simplified analytical formulations, such as those relying on spatial autocorrelation functions (ACF) between voxel pairs [144], as well as projection-domain non-parametric bootstrap techniques that resample list-mode or sinogram data [145], [146], [147], [148]. While these approaches are theoretically well established, their practical applicability is often limited. For example, projection-domain bootstrap methods are computationally demanding and have restricted capability to generate a sufficiently large number of bootstrap realizations, especially for dynamic studies and modern scanners employing iterative reconstruction algorithms.

In recent years, our group has developed an image-domain bootstrapping technique that eliminates the need for repeated reconstruction and enables efficient uncertainty estimation [149], [150], [151]. This approach has also been extended to quantify uncertainty in parametric imaging for LAFOV PET, demonstrating feasibility and scalability for whole-body dynamic datasets [57]. Meanwhile, classical statistical frameworks such as Bayesian inference have also been explored for uncertainty estimation [53], and more recently combined with deep learning for application to LAFOV PET systems [152]. These developments remain at an early stage and require further validation under realistic clinical conditions to ensure robustness, interpretability, and generalizability.

## 4 Clinical applications

In this paper, we do not aim to provide a deep discussion of specific clinical applications of LAFOV PET across different disease domains. Instead, our primary focus is on dynamic PET acquisition protocols and quantitative strategies that have the potential to facilitate the clinical translation of parametric imaging, such as abbreviated dynamic scanning protocols and dynamic low-dose approaches. For readers interested in application-oriented perspectives, we refer to dedicated literature in oncology [49], [153], cardiology [154], and neurology [155], as well as several comprehensive reviews that provide a broader clinical perspective on PET imaging [156], [157].

### 4.1 Abbreviated dynamic scans

**Fig. 5:**
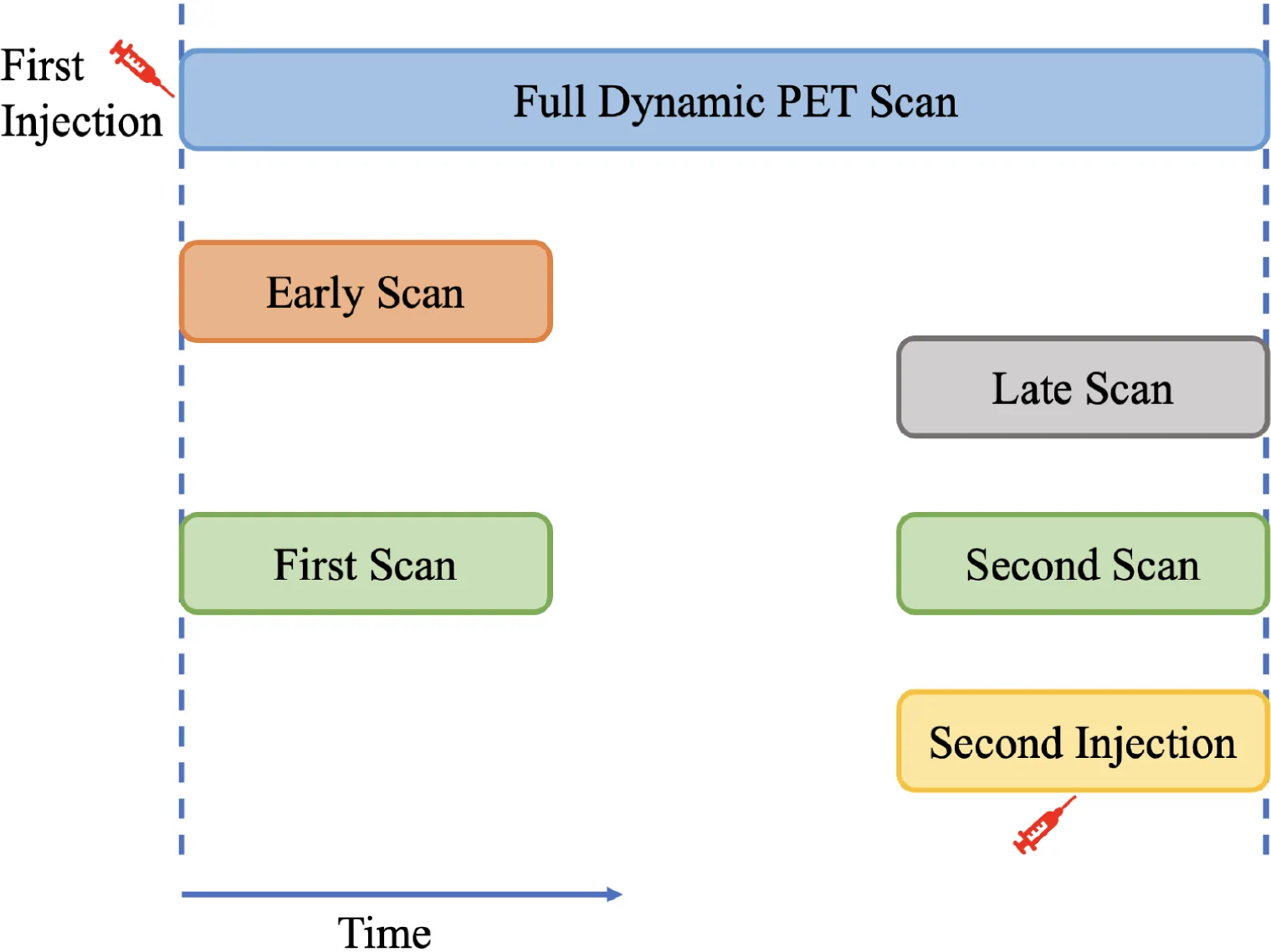
Protocols for the abbreviated dynamic PET scans.

The primary barrier hindering the adoption of parametric imaging in clinical practice is the excessively long duration of dynamic scanning, which significantly limits patient throughput, compromises patient comfort, and escalates operational costs. Shortening acquisition while preserving quantitative fidelity is therefore essential to make parametric imaging clinically viable [7]. Leveraging the high sensitivity of LAFOV PET, a range of abbreviated dynamic ^18^F-FDG protocols including early scan, late scan, dual-scan, and dual-injection approaches have been explored to accelerate workflows, as illustrated in Fig. 5.

(1) Early scan: current explorations of early-scan protocols are mainly based on compartmental modeling and Patlak graphical analysis. Feng et al. showed that parameters estimated from just the first 90 s, especially $K_{1}$ and $V_{B}$, agree closely with those from a full 1 h acquisition on the uEXPLORER system [67]. Liu et al. further demonstrated that when all kinetic parameters are considered, dynamic ^18^F-FDG scans can be shortened to the first 30 min with minimal loss of accuracy [158], [159]. With advanced data-driven techniques, such as response extension and machine learning, acquisition windows may be reduced even further, approaching around 15 min [160], [161].
(2) Late scan: most studies accelerate dynamic acquisitions by applying the Patlak model to truncated late-time windows, typically coupled with PBIF. This approach yields accurate $K_{i}$ from shortened datasets, cutting scan time by a factor of 2–3 [124], [162], [163]. When combined with denoising (e.g., non-local-mean filter) [56], [164] or deep learning [123], [132], [133], the acquisition duration can be minimized even further to as little as 10 min.
(3) Dual-scan: early only or late only protocols may miss parts of the kinetics, whereas dual-scan approach involves acquiring early and late time frames separately to comprehensively capture the full kinetic profile. However, this method necessitates precise image registration between the two scans to ensure alignment and accuracy [79], [165], [166].
(4) Dual-injection: Wu et al. also proposed a dual-injection protocol, comprising a single scanning period from 50 to 60 min after the initial injection, with a second injection administered at 56 min. This dual-injection approach exhibits strong practical feasibility, making it particularly promising for clinical use [79].

### 4.2 Multi-tracer protocols

Unlike the dual-injection protocols discussed above, which primarily aim to shorten dynamic PET scanning durations for a single tracer, multi-tracer injection protocols involve the administration of two or more tracers within a single imaging session to provide a more comprehensive characterization of tissue function [167], [168], [169], [170], [171], [172], [173], while also offering potential advantages for patient selection in radioligand therapy by combining a target-specific tracer with ^18^F-FDG to verify that all metastatic lesions are adequately targeted [174], [175].

**Fig. 6:**
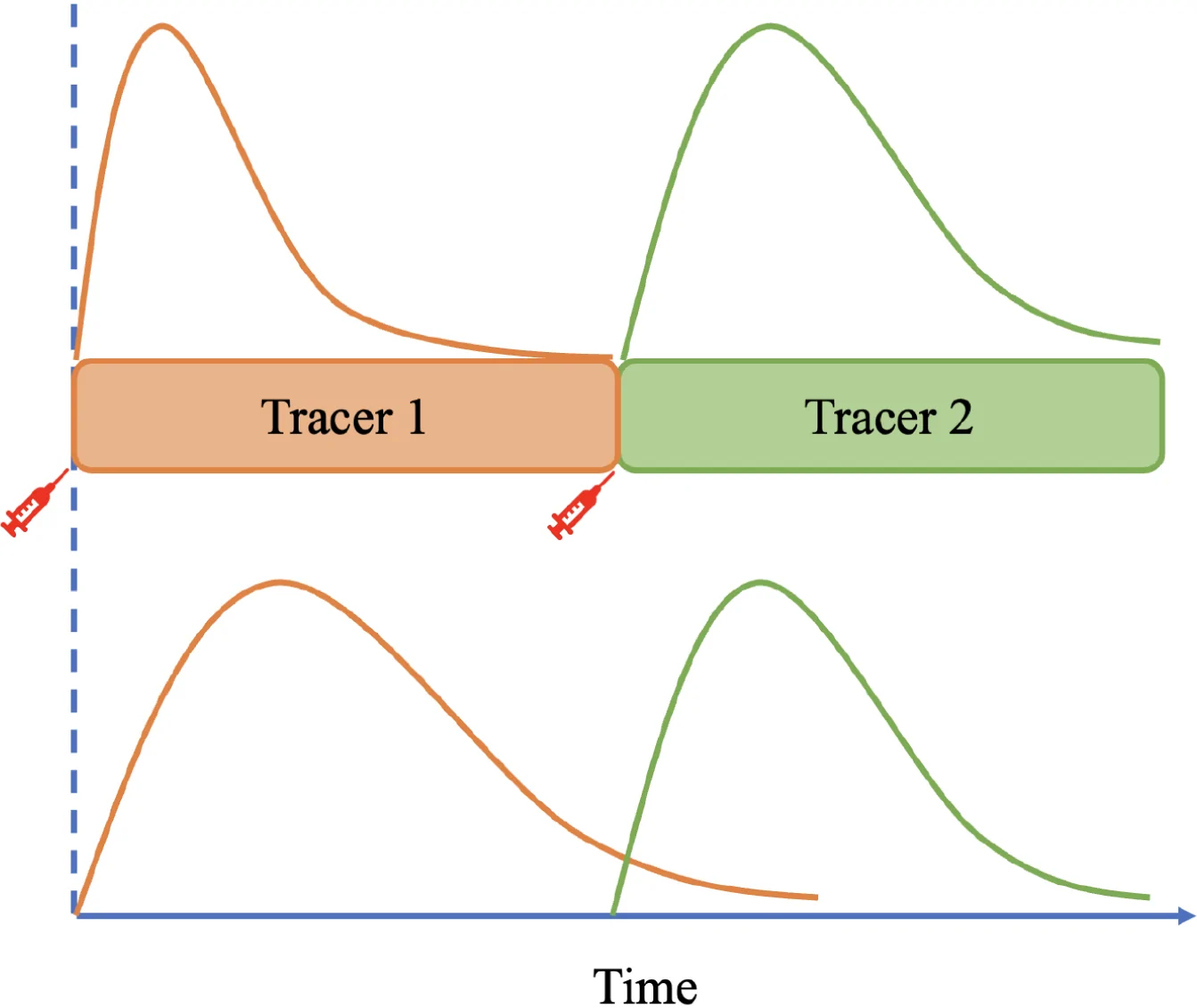
Conceptual illustration of multi-tracer injection protocols in dynamic PET. Top: scenario without overlap between tracers; bottom: scenario with overlap between tracers.

Recent studies using LAFOV PET systems have begun to explore such multi-tracer designs. Owing to differences in physical half-lives and kinetic behaviors among tracers, two practical scenarios (Fig. 6) arise:

(1) With overlap: temporal overlap between tracers may occur, with residual activity from the preceding tracer contaminating subsequent measurements. In this case, the primary methodological challenge lies in effective signal separation, which typically requires dedicated tracer separation or decomposition algorithms [48], [64], [176], [177].
(2) With little/no overlap (e.g., sufficient gap or short-lived tracers): the focus shifts to efficient joint analysis of the multi-tracer data. Our previous work has demonstrated the feasibility of a combined-analysis approach for such settings [90], which are expected to be advantageous in the LAFOV context, especially for studies investigating coupled perfusion and metabolism [178].

### 4.3 Dynamic low-dose imaging

Public static low-dose LAFOV PET datasets are now available [179], and most studies use deep learning to recover standard-dose image from ultra-low-dose acquisitions [180], [181]. But work specifically on dynamic low-dose LAFOV PET is still scarce. Muller et al. explored deep-learning denoising (DL-DN) to enhance quantitative accuracy in dynamic low-dose PET, highlighting the promise of data-driven methods for maintaining reliable kinetics under reduced counts [182].

**Table 3:** Recent software tools for dynamic PET studies (developed since 2020), with the inclusion of PMOD as a widely used commercial platform.

Software	Vendor/Developer	Commercial vs. Free	Ref.
PMOD	PMOD Technologies LLC	Commercial	
uKinetics	United Imaging Healthcare	Commercial	[80]
Multiparametric PET Suite AI	Siemens Healthineers	Commercial	[183]
DynamicIQ	GE HealthCare	Commercial	[184]
kinfitr	Karolinska Institutet	Free	[185]
NiftyPAD	University College London	Free	[186]
EMATA	University of Padova	Free	[187]
KinetiX	DMU SMART IMAGING	Free	[188], [189]
TURBO Toolbox	Turku PET Centre	Free	[190]
pyPatlak	Humanitas University	Free	[191]

### 4.4 Novel tracer and drug development

By enabling simultaneous whole-body kinetic quantification with improved sensitivity and temporal resolution, dynamic LAFOV PET combined with parametric imaging facilitates a deeper understanding of tracer biodistribution, metabolism, and target engagement across diverse physiological systems. A major application of LAFOV PET is the development of novel tracers, which inherently requires dynamic acquisitions over time determined by the tracer’s biological behavior. A growing number of such studies have been conducted using different classes of tracers, as summarized in Table 1. For newly developed tracers, where the underlying physiological mechanisms may be unclear or only partially understood, data-driven approaches that impose minimal prior assumptions, such as non-parametric or model-flexible methods, are particularly well suited.

Beyond tracer development, dynamic LAFOV PET has also demonstrated significant value in early-phase drug evaluation, offering noninvasive insights into pharmacokinetics, pharmacodynamics, and preliminary therapeutic efficacy. In particular, LAFOV PET has begun to touch upon the exploration of innovative radiopharmaceuticals, encompassing a wide range of molecular classes, including small molecules, peptides, nanobodies, and other biomolecules [27], [28], [29], [30], [31], [32], [34], [35], [38], [192]. For a comprehensive overview of the role of LAFOV PET in novel tracer development and drug evaluation, we refer the reader to several recent review articles [193], [194], [195].

## 5 Software

Software serves as a crucial bridge between clinical practices and advanced methodologies in dynamic PET imaging. Given the substantial ongoing efforts in this field, a wide range of commercial and open-source software tools are currently available for parametric imaging.

PMOD software (PMOD, Zürich, Switzerland) is one of the most widely used software packages for PET image analysis. Its Kinetic Modeling Tool (PKIN) module includes nearly all commonly used quantitative models and enables kinetic analysis through a graphical user interface without the need for any programming. Despite its comprehensive functionality and ease of use, PMOD is associated with relatively high licensing costs. In addition, several vendor-specific commercial tools, such as uKinetics (United Imaging Healthcare), are available and are often installed as part of scanner-specific software environments. An overview of the key features of commercially available hardware and software solutions is summarized in [183].

The NMMItools webpage (https://nmmitools.org), developed by Karakatsanis, provides a valuable platform of the available resources, with a particular emphasis on open-source software. Wang et al. also compiled a comprehensive table listing different software options for kinetic modeling and parametric imaging in their review paper [16]. However, many open-source repositories have become inactive, and a large proportion of existing tools are not optimized for dynamic LAFOV PET studies, particularly with respect to handling ultra-large datasets and performing accurate voxel-wise TAC fitting.

We have summarized the most recent software packages developed in the past five years, particularly those specifically tailored for LAFOV PET parametric imaging, as presented in Table 3. The continued emergence of more open-source dynamic datasets and software is both expected and essential to accelerate validation, benchmarking, and eventual clinical adoption of parametric imaging, thereby further propelling advancements in this area.

## 6 Conclusion

Dynamic LAFOV PET, as an advanced technology with enhanced sensitivity and resolution, offers unprecedented opportunities for precise whole-body quantification. This review has highlighted the key technical challenges and presented methodological solutions. These approaches, whether applied independently or in combination, pave the way for robust parametric imaging tailored to LAFOV datasets. Clinically, they enable innovative protocols like short-scan, low-dose, and multi-injection studies, as well as accelerating novel tracer and drug development. Despite promising progress, barriers such as limited datasets, software availability, and standardization persist. Future efforts should focus on advanced analysis techniques, open-source resources, multi-center validation to fully release LAFOV parametric imaging’s potential in precision medicine, ultimately improving diagnostic accuracy, treatment monitoring, and patient outcomes.

## CRediT authorship contribution statement

**Fengyun Gu:** Writing – original draft, Methodology, Formal analysis, Conceptualization. **Clemens Mingels:** Writing – review & editing, Resources, Data curation. **Robert Seifert:** Writing – review & editing. **Federico Caobelli:** Writing – review & editing, Data curation. **Ali Afshar-Oromieh:** Writing – review & editing, Validation. **Axel Rominger:** Writing – review & editing, Supervision. **Finbarr O’Sullivan:** Writing – review & editing, Supervision. **Kuangyu Shi:** Writing – review & editing, Supervision, Conceptualization.

## Declaration

The local Institutional Review Board approved the study (KEK 2019–02193), and written informed consent was obtained from all patients. The study was performed in accordance with the declaration of Helsinki.

## Declaration of competing interest

The authors declare the following financial interests/personal relationships which may be considered as potential competing interests: Robert Seifert has received a travel grant from Boehringer Ingelheim Funds and a research grant from Else Kröner-Fresenius-Stiftung, as well as travel support and speaker honoraria from Novartis and Boston Scientific, outside the submitted work.
